# Supplementary material for: Are carbon emissions trading and green financial instruments synergistic? -Comprehensive quantitative research based on content analysis
Source: PLoS One. 2024 Mar 7;19(3):e0298601. doi: 10.1371/journal.pone.0298601 (PMC10919601; doi:10.1371/journal.pone.0298601)
Supplement: S1 Data — (ZIP) [file pone.0298601.s001.zip › Data/地级市Z_F工作报告.zip!/─┌├╔╣┼/░n═╖╩╨/└┤╫╘╣1⁄2╓┌║┼í╛┬φ┐╦╩2╛▌═°í┐_2010.docx]

各位代表：

　　现在，我代表市人民政府向大会作工作报告，请予审议，并请市政协各位委员提出意见。

　　一、2009年工作回顾

　　2009年是我市经受重大考验并取得显著成绩的一年。在自治区党委、政府和中共包头市委的领导下，在市人大和市政协的支持监督下，我们深入落实科学发展观，坚决贯彻中央、自治区扩内需保增长的各项政策，采取了一系列解近忧谋长远的有力措施，积极应对国际金融危机带来的严重影响，较好地完成了市十三届人大二次会议确定的工作任务，实现了经济社会健康快速可持续发展。

　　──综合实力迈上新台阶。预计全年地区生产总值实现2160亿元左右，增长17.5%左右（根据第二次经济普查修订数据预计），继2006年跨过千亿元大关之后，三年时间再增千亿元总量。财政总收入完成244.2亿元，增长19%。

　　──拉动内需实现新突破。年初确定的592个投资5000万元以上重点项目全部开复工，其中竣工项目达到312个。全市固定资产投资完成1500.3亿元，增长38.9%；社会消费品零售总额实现617.7亿元，增长19%。

　　──人民生活得到新提高。城镇居民人均可支配收入达到23089元，农牧民人均纯收入达到7826元，分别增长10.7%和10.6%；城镇居民人均消费性支出达到18950元，农牧民人均生活消费支出达到5522元，分别增长16.6%和11.2%；全市城镇新增就业5.58万人，城镇登记失业率为3.88%。为人民群众兴办的20件48项好事实事项目全部完成。

　　──节能减排取得新成效。预计单位生产总值能耗降低7%以上，单位工业增加值能耗降低10%以上，二氧化硫减排2300吨以上，化学需氧量减排100吨以上，圆满完成了自治区下达的节能减排指标。空气质量二级以上良好天数达到308天，比上年增加13天。

　　──城市形象呈现新亮点。2009年我市获得“全国民族团结进步模范集体”、“建国60周年中国城市发展代表”、“国家科技进步示范城市”、“全国社会治安综合治理优秀地市”、“实施畅通工程模范（一等）管理城市”等多项荣誉称号，100个迎接新中国成立60周年献礼项目如期竣工，文明城市建设成果继续巩固和发展，城市品位和形象进一步提升。

　　一年来，我们主要做了以下几方面工作。

　　（一）坚定不移地推进工业强市战略，工业经济保持平稳较快增长。坚持把保工业作为保增长促发展最紧迫、最关键的任务，下大力促投资、扶企业，推动结构调整、产业升级，增强了工业经济的总体竞争力。全年规模以上工业增加值完成904亿元，增长24.5%；全部工业增加值首次突破千亿元，增长20%以上。

　　抓项目促投资力度加大。全年新建和续建了241个5000万元以上工业重点项目，完成投资711.3亿元，增长37%。包钢通过实施一批优化产业结构的项目，实现了年产钢1000万吨的重大突破。北奔重汽新型驾驶室、华锐风电、吉泰铝型材等99个项目相继竣工投产，众翔轻型飞机、东方光大光纤生产线、中复合兆瓦级风机叶片生产线、亚新隆顺100万吨稀土合金钢等项目取得重大进展。全市在建及已投产的风电项目达到200万千瓦以上，其中50万千瓦并网发电。巴音140万千瓦风电场特许权规划获国家发改委批复，打造国家级风电动力城迈出重要步伐。神华煤化工项目累计完成投资117亿元，煤化工产业在包头即将形成。光伏产业发展取得重要突破，山晟集团多晶硅试产成功。镁产业发展势头良好，引进实施镁及合金项目7个，产能达到8万吨。

　　支持企业渡难关取得积极成效。为应对国际金融危机，出台了一系列扶持政策，帮助企业解决运行中的资金、能源、原料供应和产品销售等问题。全年为220户企业提供贷款贴息资金1.08亿元，其中为工业企业补贴5163万元。争取国家扩大内需资金11.4亿元，中小企业贷款余额达到262.38亿元，比年初增加20.64亿元。积极落实大用户直购电政策，帮助包铝和6户稀土企业降低了生产成本。协调铁路部门帮助企业缓解运力紧张局面，包钢、东方希铝等重点企业产品发运量同比增加30多万吨。实施铁精粉购进价格补贴，推动包钢与我市铁精粉生产企业建立购销合作关系，累计购销铁精粉400万吨，促进了山北地区相关企业的生产经营。保工业、保增长各项政策措施的落实，为全市企业渡难关、再发展创造了条件。全年新增规模以上中小企业101户，累计达到585户。2009年初136户停产半停产企业，有121户恢复生产，企业效益逐步好转。

　　优势特色产业进一步优化升级。钢铁、铝业、装备制造、电力、稀土等产业全年实现增加值732.8亿元，对工业经济增长的贡献率达到87.8%。其中，装备制造业实现增加值194.6亿元，增长62%，对工业增长的贡献率为38.1%，成为拉动工业经济增长贡献最大的产业。钢铁、铝业、电力、稀土等产业结构不断优化，发展后劲进一步增强。

　　园区建设扎实推进。稀土高新区及包头装备制造业园区、特钢产业园区、九原工业园区、土右新型工业园区等9个重点工业园区完成基础设施建设投资14亿元，收储可利用土地1.84万亩，发展空间进一步拓展，服务进一步优化，产业集聚效应进一步显现。全年入园的5000万元以上项目达到165个，完成投资430亿元。入园企业累计达到1267户，完成工业总产值1700多亿元，增长20%以上。

　　自主创新能力不断增强。组建重型汽车产业技术创新战略联盟，技术研发水平进一步提高。北重大口径厚壁无缝钢管垂直挤压设备研发成功，百米高速钢轨等重点项目关键技术攻关取得重大突破，并产生良好效益。2009年，全市国家级高新技术企业达到45家，占自治区的58%，高新技术企业实现产值258亿元，占自治区的50%以上。市级以上创新型试点企业新增20家，包钢成为我市首家国家级创新型企业。稀土高新区进入国家首批新型工业化产业示范基地行列。

　　（二）坚定不移地推进现代服务业发展，第三产业实现提质增效。积极发挥消费的拉动作用，不断提高服务业发展水平，预计全年增加值突破900亿元，增长16%以上。

　　服务业项目投资实现重大突破。全年实施服务业重点项目308个，占全部592个重点项目的52%，完成投资458.7亿元，增长22.4%。东源国际、帝豪天下等项目竣工并部分投入使用，东方红广场、万达广场、金基广场等大型商业项目加速推进，成为拉动我市经济增长的新亮点。

　　消费市场繁荣活跃。把扩大消费需求作为应对危机的重要举措，实行了一系列引导消费的政策。为促进住房消费，财政投入2647万元，对居民购买120平方米以下普通商品住房给予补贴，受益居民达2.6万户。全市新建商品房销售、二手房成交量分别达到37599套和21795套，增长106.6%和212.7%。汽车销售达到5.08万辆，增长18. 3%。大力推动“家电下乡工程”、“汽车下乡工程”，全面实施“万村千乡市场工程”和“双百市场工程”，扩大了农村牧区消费。全市家电下乡销售网点达到120家，累计销售家电产品4.04万台，实现销售额6534.25万元。新建、改造农家店110家，建设配送中心5个，覆盖了80%以上的行政村。

　　传统服务业稳步发展。积极推动批发、零售、餐饮等产业扩大规模、提高质量，王府井百货、包百、华联等企业进一步发展壮大，友谊蔬菜批发市场、沙尔沁蔬菜副食品交易市场进入国家“双百市场工程”行列，永盛成连锁超市开拓全区市场实现新突破，小肥羊、小尾羊成为餐饮行业和“群羊产业”的领头羊。

　　现代服务业发展持续升温。物流业发展呈现新亮点，土右旗煤炭物流基地、新奥蒙华化工物流中心建设进展顺利，古城湾环保型煤炭装车基地全年实现装车量452万吨。目前，全市共有物流注册企业580多家，年营业额5000万元以上的达到75家。我市跻身全国流通领域现代物流示范城市行列，成为全国17个区域性物流节点城市之一。金融对经济增长的促进作用显著增强，全市本外币各项存款余额1502.84亿元，较年初增加288.18亿元，增长23.73%；各项贷款余额1073.05亿元（包括企业从外地金融机构贷款），较年初增加259.31亿元，增长31.87%。招商银行、浦东发展银行、中信银行分支机构在我市正式运营。包商银行跨地区发展取得重大突破。全市批准筹建小额贷款公司66家，注册资金69.48亿元，开业50家，累计贷款43.61亿元。旅游业平稳有序发展，全年旅游接待人数526.8万人次，旅游总收入95亿元。信息产业规模不断扩大，稀土高新区软件园入驻企业累计达到128家，实现销售收入30.5亿元，增长38.6%；实现利税2.33亿元，增长36%。会展业发展步伐加快，2009年我市成功举办了中国包头·稀土产业发展论坛、中国新型煤化工国际峰会、第三届中国制造业论坛、国际风能技术与投资高峰论坛、中国塑料产业论坛等大型会展活动，包头的知名度和影响力进一步扩大。

　　（三）坚定不移地推进“三农三牧”工作，促进了农牧业发展和农牧民生活水平的提高。始终把解决“三农三牧”问题作为全市工作的重点，不断加强农牧业基础设施建设，全面启动城乡一体化发展战略，全市呈现出农牧业较快发展、农牧民持续增收的良好局面。预计第一产业实现增加值55亿元，增长5.9%。

　　农牧业结构更加优化。加快种植业结构调整，全市马铃薯、蔬菜种植面积分别达到101.9万亩和15.9万亩，增加10.7万亩和2.7万亩。在山北地区遭受严重旱灾的情况下，粮食产量达到100.6万吨，创历史新高。设施农业快速发展，滴灌、覆膜、喷灌技术推广力度进一步加大，大型灌区节水改造工程稳步推进。山北地区设施马铃薯面积达到26万亩，增加20万亩。大力推进牲畜规模化饲养，肉羊和奶牛养殖规模化水平分别达到50%和70%，提高15个和30个百分点。

　　农牧业产业化深入推进。全市规模以上农牧业产业化加工企业和流通企业全年实现销售收入170亿元和32亿元，分别增长11%和6%。市级以上龙头企业增加18户，达到75户。农畜产品行业中国驰名商标新增1个，达到8个。全市农牧民专业合作经济组织增加115家，达到252家。加入合作组织的农牧户增加4000户，达到9600户。

　　农村牧区基础设施建设步伐加快。切实加大支农支牧力度，全年投入财政资金8.6亿元，增长30.1%。全市新农村新牧区建设试点村共投入资金8400万元，第二批试点村80%以上实现了安全饮水、通油路和建有卫生室等目标。农业装备水平不断提高，全市农业综合机械化水平预计达到75%以上，达茂旗在自治区率先基本实现农业机械化。

　　农牧民实现持续增收。全面落实国家对农牧民的各项补贴政策，及时足额发放粮食直补、农资综合补贴、良种补贴和农机具补贴2.09亿元。全市农牧民家庭经营性纯收入和外出务工收入人均达到4415元和942元，分别增长6.7%和12.5%。加大扶贫开发力度，争取各类扶贫资金1.2亿元，实施整村推进、产业化扶贫、移民扩镇项目37个，项目区的农牧业综合生产能力和可持续发展能力明显增强。集中社会力量帮扶固阳县工作进展顺利，实施项目182个，落实资金和物资8073万元，社会扶贫覆盖固阳县104个行政村。

　　（四）坚定不移地推进城市建设，生态环境明显改善。遵循城市发展规律，强化基础设施建设，提高管理水平，城市现代化步伐进一步加快。

　　城市承载功能显著提升。全年实施城建重点项目169个，完成投资210多亿元。新图书馆、少年宫、体育场、体育馆等重点项目取得重大进展。民族东路改造、机场高速立交桥西延等道路改扩建工程顺利完工，全市新增道路面积130万平方米，人均道路面积达到12平方米，增加0.34平方米。锦林公园、稀土公园等一批园林绿化项目投入使用，新增绿地面积190万平方米，全市建成区绿化覆盖率达到39%。组织实施城市中心区供水排水管网建设、城区污水管网改造、城市集中供热节能改造等一批基础设施工程。城市供水能力由每日51万立方米提高到100万立方米以上，燃气普及率达到90%，集中供热普及率达到84%，生活污水处理率达到82%，再生水利用率达到30%。累计完成212万平方米住宅小区保温改造，建筑节能工作取得新成效。包头医学院、体育场、赛立特尔搬迁改造等项目有序实施，金融商务区、文化艺术中心等一批标志性工程全面启动。旧街坊、旧小区、“夹心房”和城中村、城乡结合部改造工程取得明显进展，人居环境和质量进一步提升。新都市区、滨河新区、昆区南部区、青山北部区、石拐南部区建设步伐加快，城市框架进一步拉大，中心城区建设用地规模达到212平方公里。公路、铁路、民航建设水平不断提高，市区到外五旗县区将逐步实现高等级公路连通，包头至西安双线电气化铁路部分开通，包头机场航线增加至16条，旅客吞吐量突破100万人次。

　　城市管理和环境保护力度加大。继续巩固全国文明城市创建成果，开展城市管理综合执法，清理违章建筑，整治交通秩序，市容市貌有了新的改观。建筑工程管理进一步加强。深入开展创建国家环保模范城市行动，积极推进排污整治工作，关停了一批落后产能，开工建设土右旗、白云矿区、万水泉工业园区和包头铝业产业园区等地区和园区的污水处理厂。组织实施了“三片一线”等重点区域、重点行业、重点企业的环境整治，加强大青山沿线砂场、料场整治和南绕城煤场清理工作，城市环境继续改善。

　　生态建设全面推进。“绿水、青山、大草原”三大生态工程深入实施，小白河蓄滞洪区工程进展顺利，黄河湿地路坝一体工程全线推进，大青山南坡10万亩绿化成效明显，京藏高速公路两侧景观林带初步形成，围封禁牧全面实施，区域生态环境明显好转。城郊结合部绿化完成8985亩，投资1亿元的碳汇造林项目已获批复，生物质能源综合项目正式启动，大青山生态应急水源工程顺利实施。

　　（五）坚定不移地推进改革开放，发展活力不断增强。坚持以改革增活力，以开放促发展，着力突破体制机制制约，增强了科学发展的内生动力。

　　重点领域改革不断深化。加快政府职能转变，进一步加强社会管理、公共服务。全市政府机构改革扎实推进，事业单位岗位设置管理和义务教育绩效工资制度改革稳步推进，国库集中支付、部门预算全面实施。市属国有企业体制改革基本完成，国有资产监管体制和运营机制继续完善，为90户中小企业减债12.7亿元，鹿王、包头宾馆、青山宾馆等一批改制企业焕发出新的生机与活力。启动医疗体制改革各项工作。农村牧区综合改革有序推进，集体林权制度改革取得新进展，水利工程管理体制改革全面完成。建立了银政企合作协调机制和金融工作激励机制，有力地促进了金融业的发展。

　　对外开放进一步扩大。广泛开展招商引资，组织了面向韩国、香港和长三角、京津冀的大型招商引资活动。全市引进国内区外资金342.8亿元，增长17.76%，引进亿元以上项目67个。全市实际利用外资达到9.49亿美元，增长14%。受国际金融危机影响，我市外贸进出口下降45.2%。满都拉口岸升格为国家对外开放一类口岸，货运通道、综合业务楼、联检大楼改扩建等基础设施项目顺利推进。援建蒙古国的杭吉口岸联检楼、公路等工程取得积极进展。我市与国内外地区、城市的协作日渐增多，年内与120多个城市开展了友好往来与合作。

　　（六）坚定不移地保障和改善民生，人民群众得到更多实惠。加快以改善民生为重点的各项社会事业发展，促进了人民群众安居乐业，维护了社会和谐稳定。

　　就业和社会保障工作扎实开展。积极应对国际金融危机带来的就业压力，制定出台了减轻企业负担相关政策，基本实现了多数困难企业不裁员、不降薪。全力实施以创业带动就业和失业预警等政策措施，促进高校毕业生、农村牧区富余劳动力和就业困难人员就业取得明显成效，保持了就业形势基本稳定。全年新增大学生就业1.4万人，下岗失业人员再就业19963人，安置困难人员就业4055人，帮助1660户“零转移家庭”的3463人实现就业，农牧民外出就业22.25万人。启动实施了包头市城乡居民基本养老保险、原单位职工补办基本养老保险、私营企业员工和个体劳动者补办基本养老保险以及城镇职工基本医疗保险补充办法5个政策性文件，养老、医疗保险政策实现对城乡居民全覆盖。社会保障范围稳步扩大，城乡居民基本养老保险参保25.2万人，城镇居民基本医疗保险参保45.1万人，新型农村牧区合作医疗参合率达到97.45%。城乡社会救助体系逐步完善，社会救助保障水平继续提高。城镇居民低保标准提高30元，达到每人每月310元。农牧民低保标准提高200元，达到每人每年1500元；低保补助水平提高180元，达到每人每年1180元。城乡低保标准居自治区首位，高于全国平均水平。企业退休人员养老金月人均增加125.6元，达到1326元，高于全国和自治区水平。住房保障工作深入推进，全年新建续建经济适用住房156万平方米，廉租住房保障资金支出2169万元，15338户低保家庭享受了廉租住房补贴。石拐棚户区和包钢尾矿坝周边五村搬迁改造工程全面完成，1.5万户4万多居民将迁入新居。

　　科技、教育、文化、卫生等社会事业迅速发展。大力实施“科教兴市”战略，以企业为主体、政府引导的科技工作投入机制不断完善，全年投入研发经费14.3亿元，增长20%。深化产学研合作，组织实施了资源综合利用与节能减排新技术推广应用、科技惠民等科技专项和一批产学研合作项目，稀土产业技术创新联盟被列为自治区级产业技术创新联盟试点，科技进步对经济社会发展的推动力显著增强。人才资源开发力度加大，引进国内外人才和智力工作成效明显。加大对城乡义务教育的投入，基础教育继续保持自治区领先水平。民族教育得到优先重点发展。实施职业教育资源整合，对全市现有的中高等职业学校进行调整合并，启动了职业教育基地建设。中小学校舍安全工程开始推进。加快文化大市建设步伐，推进文化基础设施建设和文化产业发展，传媒大厦、广播电视采编制作播出中心等一批项目开工建设。成功举办了首届中国包头秦长城热气球节和首届内蒙古二人台艺术节。创作排演了《西口好人》等一批精品剧目。体育事业稳步发展，2009年全国航海模型比赛等6项全国赛事在包头顺利举办，全市第十一届运动会以及首届少数民族传统体育运动会取得圆满成功。为266个行政村安装了体育健身器材。完成106个农村牧区广播电视数字村建设任务。建成市肿瘤医院等3家医院病房楼，新建改建了一批社区卫生服务中心、乡村卫生室。为20万40岁以上的农牧民免费体检。开展“健康包头行动”，10万多群众受益。人口和计划生育工作继续走在自治区前列，人口自然增长率为4.91‰。

　　社会管理工作有效加强。不断完善各类应急预案和应急体系建设，投入1920万元，采取有力措施防控甲型H1N1流感取得胜利，保证了人民群众的生命健康安全。食品药品监管工作继续加强。认真落实安全生产责任制，深入开展社会治安综合治理，维护了社会稳定。加强精神文明建设，广泛开展有地方特色的文明企业、文明社区、文明市场、文明窗口等创建活动，城乡居民的综合素质进一步提高。全面落实党的民族宗教政策，制定实施了一系列符合我市实际的措施，各民族团结进步、繁荣发展的大好局面进一步巩固。人事、审计、司法、统计、人防、气象、防汛、地震、工商、质量技术监督、新闻出版、侨务、外事、老龄、双拥、史志、档案、仲裁、政府法制等各项工作均取得了新成绩，为构建和谐包头做出了新贡献。

　　（七）坚定不移地推进政府自身建设，行政效能进一步提高。坚持依法行政，全面贯彻落实行政执法责任制。执行市人大及其常委会的决定，并及时向市政协常委会通报情况。全年办理人大代表建议131件、政协提案419件，办理工作质量和效率有了新提高。充分利用政府网站、新闻媒体、市长热线等平台，推进了政务公开。全面清理行政审批事项，进一步规范了审批主体、条件、程序、办理期限和收费标准。加强政府廉政建设，充分发挥行政监察、审计监督作用，加大对商业贿赂和损害人民群众利益不正之风的治理力度，严肃查处了一批违法违纪案件。高度重视信访工作，开展了领导干部大接访等活动，群众诉求渠道进一步畅通，一些信访积案得到妥善处理，维护了人民群众的合法权益，信访工作局面不断好转。

　　各位代表，2009年，是我市经济平稳较快增长、民生继续改善、社会全面进步的一年。我们深切地体会到，在工作中，必须把发展作为第一要务，坚持科学发展、富民强市；必须把实现人民幸福作为第一追求，坚持以人为本、亲民惠民；必须把建设和谐社会作为第一责任，坚持统筹兼顾、全面进步；必须把解放思想作为第一动力，坚持改革开放、开拓创新；必须把谋事干事成事作为第一准则，坚持凝心聚力、真抓实干。这是我们推动经济社会又好又快发展、不断提高人民群众生活水平的根本保证。一年来，全市人民同舟共济，攻坚克难，建设富裕民主文明和谐新包头迈出了坚实步伐。这是中共包头市委正确领导的结果，是市人大、市政协和社会各方面支持与监督的结果，是全市人民团结奋斗的结果。在此，我谨代表市人民政府，向全市各族人民，向各位人大代表、政协委员，向各民主党派、工商联、无党派人士、人民团体、社会各界，向中央和自治区驻包单位，向驻包部队、武警官兵，向所有参与、关心、支持包头建设和发展的同志们、朋友们，表示崇高的敬意和衷心的感谢！

　　在看到成绩的同时，我们也清醒地认识到，前进道路上还存在不少困难和问题，主要是我市结构性矛盾依然比较突出，经济发展的质量和效益还不高，节能环保面临的问题较多，调整经济结构、转变发展方式任务还很繁重；城乡发展不平衡，农村牧区发展相对滞后；社会保障压力较大，就业形势不容乐观，改善民生任务还很迫切；在金融危机影响没有根本消除的情况下，不稳定、不确定因素依然较多；转变政府职能、提高工作效率，还有很多工作要做，等等。对这些问题，我们将采取更加有效的措施，认真加以解决。

　　二、2010年主要工作

　　2010年是实施“十一五”规划的最后一年，也是我市在应对危机中实现新跨越的关键一年。面对依然严峻的宏观形势，我们要坚定发展信心，增强忧患意识和责任意识，千方百计巩固和发展全市经济企稳回升的大好局面，确保圆满完成“十一五”规划奋斗目标。

　　今年政府工作的总体思路和主要任务是：坚持以科学发展观为指导，认真贯彻中央、自治区经济工作会议精神，按照中共包头市委的部署，深入实施科学发展、富民强市战略，以转变发展方式为主线，以经济结构战略性调整为重点，以项目建设为抓手，大力推进科技进步和产业升级，增强发展的有效性；大力推进城乡一体化，增强发展的协调性；大力推进体制机制创新和对外开放，增强发展的驱动力；大力推进生态文明建设，增强发展的可持续性；大力推进富民惠民进程，增强发展成果的普惠性，全力保持经济社会平稳较快发展，为“十二五”发展奠定更加坚实的基础。

　　综合考虑各方面因素，今年全市经济社会发展的主要预期目标是：地区生产总值增长16%左右，财政总收入增长18%，规模以上工业增加值增长20%左右，固定资产投资增长20%以上，社会消费品零售总额增长18%以上，城镇居民人均可支配收入增长13%左右，农牧民人均纯收入增长12%左右，城镇登记失业率控制在4%以内，人口自然增长率控制在5‰以内，确保完成自治区下达的节能减排指标。

　　为此，今年要着重做好以下工作：

　　（一）充分发挥老工业基地优势，扎实推进新型工业化进程。继续实施项目带动战略，促进企业规模化、产业集群化，千方百计提高工业经济发展质量和效益，实现工业大市向工业强市的转变。

　　以项目建设为抓手推动产业优化升级。围绕国家产业调整和振兴规划，加快重点项目建设，构建现代产业体系。今年要组织实施5000万元以上工业重点项目289个，力争完成投资850亿元，增长20%。加快发展优势特色产业。大力推进神华煤制烯烃、海平面PVC项目，年内要建成投产；积极推动泛海煤化工、汉龙PVC等项目建设，为塑料等下游产业发展提供有力支撑。支持包钢实施结构调整、产业延伸项目，推动华业特钢不锈钢宽厚板、国电增速器、晋西铁路车轴、泰利C型钢异型钢生产线、德顺特钢、北镁科技、亚新隆顺等一批项目早日竣工。加快实施加拿大汉维风力发电机组、江海硅铝合金、广西桂东电子铝箔、华科稀土微晶玻璃和多孔微晶玻璃项目等一批重点项目。培育战略性新兴产业。以风、光等新能源，稀土新材料，光伏电子材料，塑料光纤，风力发电设备制造为重点，深入实施无锡尚德光伏发电、包钢稀土高性能磁性材料、众翔轻型飞机等项目。研究制定扶持装备制造业发展的政策措施，加快发展配套产业，延伸产业链，形成产业基地。全年装备制造业销售收入要突破700亿元。支持生物质能源和环保产业重点项目建设，大力发展低碳经济和循环经济。继续做好项目策划，争取更多资金投入和项目支持。

　　提升工业园区综合承载能力。加强基础设施建设，完善行政、融资、技术、人才服务体系，优化园区发展环境。推动现有重点园区做大做强，支持固阳工业园区发展。进一步明确各园区发展规划定位，强化产业分工协作，实现园区优势互补和错位发展，努力将我市工业园区建设成为“一园一主业、主业特色化”的新型产业基地。加强对园区工作的考核，建立推动园区发展的激励机制，提高园区投资强度、项目密度和产业集聚度，把园区建成产业发展、企业成长、就业促进的重要平台。

　　全力支持企业发展。完善市领导联系重点企业制度，密切跟踪分析重点产业、重点企业、重点产品的运行态势，帮助企业积极应对市场变化。高度重视优势企业发展，全力支持包钢、一机、北重、包铝、中核北方核燃料元件有限公司、鹿王羊绒等企业拓展市场、扩大销售、增加效益。推动东方希铝、北重集团和电力系统企业产值突破百亿元。研究制定促进中小企业发展的政策措施，实施中小企业入园发展、上档升级、人才培养、市场开拓、政策扶持五大工程，推动中小企业向专、新、特、精方向发展，实现以小补大、以专配全，打造产业集群。以市场为导向，创造公平竞争的环境，扶持本土企业不断壮大，增强企业发展活力。加快发展非公有制经济，降低市场准入条件，只要符合中央、自治区政策，都允许民间资本进入。加强政府引导和服务，拓宽融资渠道，促进非公有制经济发展上水平、上档次。

　　增强科技对工业经济的拉动力。加快信息化与工业化融合，以信息化推动工业化。强化企业主体地位，培育自主创新能力，推进科技资源、创新要素向企业聚集，完善产学研相结合的创新体系。加大科技研发投入，大力实施企业技术创新引导工程，引进和培养高科技人才。围绕优势特色产业和战略性新兴产业，创建一批国家级、自治区级产业技术创新联盟和重点实验室。组织实施重大科技专项，加快建设高速铁路钢轨和镁合金研究中心，推动低成本高性能钢材研发等一批关键技术研发实现突破。以我市成为首批国家创新型试点城市为契机，深入开展创新型城市建设。

　　（二）大力发展现代服务业，增强消费对经济增长的拉动作用。抓住扩大内需的有利时机，按照发展提速、比重提高、结构优化的总体要求，推动服务业快速发展。

　　提升服务业现代化发展水平。落实好支持服务业发展的政策措施，优化服务业发展环境。完善包百等商务区服务功能，培育发展流通新业态，促进商贸经济的提质增效。大力发展特色街区，推动北梁明清古文化商业步行街尽快投入使用。加快东方红广场、万达广场、金基广场、燕赵大厦、华银大厦和高新区总部经济园等一批商业开发项目建设，年内要竣工投入使用。大力发展物流业，推动运输、仓储、快递等企业功能整合和服务延伸，促进传统物流企业的现代化转型。依托大城西煤炭物流和古城湾环保型煤炭装车基地、新奥蒙华化工物流中心、昕隆不锈钢产品物流中心，建设全国或区域性的大型煤炭、钢材、有色金属、工程机械、稀土、机电装备、建材、重化工产品、重型汽车交易市场，全面提升生产性物流业发展水平。抓好重点物流企业建设，培育一批具有较强辐射力的区域营销中心。积极发展会展经济，以会展中心落成为契机，力争主办一次全国性展览展示会，打造会展品牌。加强旅游景点建设，整治五当召等景区、景点周边环境，促进旅游业快速发展。

　　努力扩大消费。继续把促消费作为保增长的重要举措，保持鼓励消费政策的连续性。不断提高城乡居民特别是低收入群众消费能力，建立完善企业职工工资正常增长机制和支付保障机制，适当提高公务员津补贴，稳定收入预期。推动房地产企业优化供应结构，培育发展二手房市场和房屋租赁市场，积极引导住房消费。进一步落实好国家鼓励汽车消费的各项优惠政策，加强停车场建设和管理，推动汽车消费。鼓励城乡居民文化、旅游等消费，提升消费层次。强化消费信贷服务，完善消费设施和服务体系，优化消费环境。促进便民消费，新建和改造50家社区便民店。增加早餐经营网点，力争三年时间发展1000个，基本覆盖全市主要社区。扩大农村牧区消费，积极推进“汽车、摩托车、家电下乡”工作。深入实施“万村千乡市场工程”和“双百市场工程”，发展乡镇连锁经营，新建农家店50家，累计达到565家，覆盖90%以上行政村。

　　（三）统筹城乡协调发展，扎实做好“三农三牧”工作。在维护农牧民切身利益、促进农牧民增收和农村牧区发展的基础上，全面推进城乡一体化，推动农村牧区改革发展迈上新台阶。

　　 加快城乡一体化进程。认真实施城乡一体化规划纲要，率先推进城乡结合部治理、小城镇建设和农牧民稳定转移，努力在空间布局、产业发展、基础设施建设、公共服务、社会保障、社会管理、土地管理一体化等方面取得突破。到2011年全市城镇化率达到80%以上，农牧业人口减少到50万人。加大以工哺农、以城带乡力度，增强农牧业旗县区自我发展能力。加快土右旗中等城市建设步伐。继续举全市之力帮扶固阳县。深入实施“收缩、转移、集中”战略，妥善做好农牧民安置工作。大力推进新农村新牧区建设，进一步完善农村牧区基础设施，力争实现试点村村村通油路和安全饮水，用三年时间完成农村牧区危旧房改造任务。

　　发展现代农牧业。推进农牧业设施化、产业化、规模化，加快转变农牧业发展方式。深入实施“北薯南菜”战略，调整优化农牧业结构。加强蔬菜保护地建设，新增蔬菜保护地1.2万亩。做大做强马铃薯产业，马铃薯种植面积力争达到115万亩，其中设施马铃薯46万亩，优质脱毒种薯7万亩。强化农牧业科技支撑，大力发展节水灌溉，进一步提高农业机械化水平。充分发挥农牧业龙头企业的带动作用，加快奶牛标准化养殖小区建设，推进肉羊舍饲圈养和品种改良，提高规模化、集约化发展水平。加快推进农牧业产业化，积极引进培育奶联科技乳品等农畜产品加工和流通企业，新增市级以上龙头企业10个。

　　深化农村牧区改革。加快推进土地承包经营权有序流转，年内土地流转面积增加38万亩，达到110万亩。全面推进集体林权制度改革。大力发展农牧民专业合作经济组织，全年增加48个，达到300个。完善支农支牧资金稳定增长机制，鼓励社会资本参与农牧业开发和新农村新牧区建设。

　　千方百计提高农牧民生活水平。认真落实粮食直补、良种补贴、农机具购置补贴、农资综合补贴等各项支农惠农政策，落实好自治区出台的对牧民和牧业生产的补贴政策，落实好我市出台的围封禁牧补贴等政策，不断增加农牧民政策性收入。完善产业化利益联结机制，使农牧民从农畜产品加工转化增值中更多受益。大力发展农村牧区二三产业，拓宽农牧民就业增收渠道。积极引导农牧民有序转移，加强就业能力培训，全年培训农村牧区劳动力1.5万人，农牧民外出就业达到24万人。落实对口帮扶制度，进一步做好农村牧区扶贫工作。更加重视改善少数民族聚居地区人民群众的生产生活条件，积极扶持革命老区、边境地区和贫困地区加快发展。

　　（四）坚持改革开放，增强经济社会发展活力。推进体制创新，完善改革发展的动力机制，构建充满活力、富有效率、更加开放、有利于科学发展的支撑保障体系。

　　全面推进各项改革。积极稳妥地推进政府机构改革，进一步理顺职能，提高效率。规范政府投资行为，建立和完善投资项目绩效评审、重大项目公示及责任追究制度，健全投资项目核准制和备案制。继续深化和完善部门预算、国库集中支付、政府采购、收支两条线等各项改革，全面推进预算单位公务卡制度改革，进一步规范市级财政转移支付制度，健全和完善旗县区财政管理体制。强化国有资产监管，积极发挥国有资产运营公司的融资作用，创新融资平台。大力推进医药卫生体制改革。加快文化体制改革。深化事业单位人事制度改革，稳步实施绩效工资制度。

　　继续扩大对外开放。大力引进产业带动强、科技含量高、投资强度大、资源消耗低、符合环保要求的项目，积极吸引大公司在我市设立地区总部、营运中心、采购中心和结算中心。引导外来投资以收购、兼并、控股、参股等形式参与我市企业改革重组。加快满都拉口岸建设，全面建成我方及援建蒙古国的基础设施，推动企业与蒙方合作，扩大过货量。加快国际集装箱公司改制工作，加强包头“无水港”建设，不断拓展开放空间。完善招商引资责任体系、考评激励机制和重点项目服务机制，提高招商项目履约率、资金到位率和项目开工率。全年引进国内区外资金要增长10%以上。

　　转变外贸增长方式。抓住国际分工调整机遇，深入实施科技兴贸和品牌兴贸战略，培育一批具有自主知识产权、自主品牌的出口拳头产品，巩固传统出口市场，积极开拓新兴国际市场。加快出口贸易结构调整，推动加工贸易从简单加工制造逐步向研发、销售环节延伸。做大做强服务贸易，加快建设服务外包基地。支持有条件的企业实现跨国生产与经营。

　　（五）加大城市建设管理力度，精心打造宜居宜业城市。按照服务发展、适宜人居、环境优美的要求，加快城市现代化建设步伐，向国家生态园林城市目标迈进。

　　强化城乡规划管理。坚持以规划为龙头引领城乡建设，认真做好新一轮城市总体规划实施工作。加快完善道路、给排水、供电、供热、通讯、绿地等专项规划，完成东河区东部地区、九原区西北和东北组团、高新区拓展区等重点区块控制性详规和城区重点地段的形象设计。结合“十二五”规划编制，完善全市城乡一体化发展规划，完成全市50%以上乡镇的总体规划和40%以上村庄的规划，进一步推动城乡建设、管理的一体化。

　　大力实施城市建设项目。加快城市基础设施建设，提高城市承载能力和服务功能。今年全市拟实施城建项目140个，总投资594.1亿元，年内完成投资286.2亿元。实施“四横四纵”道路建设工程，打通青山路、站前路、哈屯高勒路和站南路，贯通包钢西路、昆河西路、沼南大道和色楞湾路，建设铁西高架路，增设哈屯高勒路与建华路互通高架桥。加快实施钢铁大街等12条道路街景综合改造项目，阿尔丁南大街等10条道路绿化、美化项目以及科学路等9条道路拓宽改造项目，努力形成路桥纵横、流转通畅、功能完备的城市交通网络，着力打造中心城区出行半小时交通圈。实施市容综合整治工程，加大东兴东出口、210国道南出口、民族东路北出口等城市出口环境整治力度。推进公用设施建设工程，改造2座水质净化厂，新建2座污水处理厂和1座污泥处理厂，改扩建1座垃圾卫生填埋场，外五旗县区城关镇要全部配套建设污水处理厂和垃圾填埋场。新建10座垃圾转运站、15座水冲式公厕。全面开展城市地下管线普查工作，实施60公里供气管网工程和90公里供热干网工程，对居住区供热和供水二次管网以及已经老化的污水管网进行改造，进一步完善市政基础设施。加强和改进城市监控系统建设。实施园林景观工程，建设奥林匹克公园、万水泉台地公园等大型公园，继续推进“百万大树绿鹿城”工程，完成城区新增大树35万棵的任务，提高人均绿地水平。建设完成“走西口”等一批具有历史文化内涵的城市雕塑，建成水景瀑布工程，形成城市景观新亮点。加快推进植物馆和规划馆建设，进一步完善城市公共文化和社会服务设施。着力推进保障性安居工程，建设经济适用住房和棚户区安置房234万平方米、廉租住房26万平方米。整治10个旧小区和50处“夹心房”。完成200万平方米既有居住建筑节能改造。按照城市化发展要求，抓好不适宜在市区发展的“城中厂”、“城中校”搬迁改造工作，实施新城村、昌福南村等8个“城中村”改造工程，改善城市环境。启动石拐棚户区改造二期工程，加快卜尔汗图镇及周边地区开发建设。支持东河区北梁地区开发改造和石拐南部区建设，全力推动新都市区、滨河新区、昆区南部区和青山北部区建设，进一步拓展城市空间，提升城市品位。

　　推进大交通网络建设。加快西南绕城高速公路、110国道包头协力气至东兴一级公路、104省道百灵庙至白云鄂博一级公路、104省道百灵庙至希拉穆仁一级公路、211省道固阳至百灵庙一级公路、市区至石拐一级公路等项目建设，建成包头北出口至固阳明登一级公路，大幅提升全市干线公路等级，提高通行能力和运输效率。全年公路交通建设计划完成投资30亿元以上。继续实施一批农村牧区公路建设项目，使全市行政村通公路率达到96%。加快建设飞机场扩建工程，力争年底前完工。进一步完善航空网络布局，增开3至4条航线，同时努力将包头机场申报为国际航空口岸机场。做好机场迁建工程和城市轻轨工程前期准备工作。包满铁路、包头至树林召高速公路黄河特大桥今年要竣工投入使用。积极支持呼包高速公路8车道扩建和时速300公里的京包快速铁路客运通道项目建设。

　　切实加强城市管理。继续完善城市管理体制，强化各地区、有关部门的责任，促进城市管理精细化。规范建筑工程管理。健全管理制度，采取有力措施，切实加强和改进居民小区物业管理。大力实施“畅通工程”，加大交通执法力度，努力使城市交通秩序进一步好转。加强对城乡结合部和拆迁改造区域的综合整治，坚决制止和依法打击私搭乱建等违法行为。进一步做好城市公共基础设施管护工作，创造安全、便捷、舒适的城市环境。

　　（六）深入推进生态文明建设，提高可持续发展水平。坚持走生产发展、生活富裕、生态良好的文明发展道路，全力创建国家环保模范城市，推动资源节约型和环境友好型社会建设取得新成效。

　　强化节能减排和环境保护。加大源头治理力度，坚决淘汰落后产能和工艺。突出抓好钢铁、铝业、电力、化工、建材等重点行业、重点领域、重点企业的节能工作，确保万元工业增加值能耗下降7.51%。健全完善节能减排监管机制，严格执行节能评估审查制度。认真落实国家产业政策，加强环境影响评价和建设项目“三同时”管理，加快重点行业脱硫改造工程，完善减排三大体系建设，确保二氧化硫排放量控制在18万吨以内，化学需氧量排放量控制在2.7万吨以内，空气质量二级以上良好天数达到312天以上。

　　持续改善生态环境。大力实施天然林保护、退耕还林、沙源治理等重点工程。深入推进“绿水、青山、大草原”三大生态工程建设，加强大青山南坡绿化工程的验收及后期管护，按照规划要求积极开展黄河湿地沿线生态恢复工作，对黄河堤坝城区重点地段北侧100米范围进行绿化、美化。完善禁牧和舍饲圈养引导鼓励政策，加大督查管理力度，巩固围封禁牧、生态恢复成果。加快推进昆河二期、东河二期、二道沙河和四道沙河综合治理工程，有效改善周边环境。抓紧实施大青山生态应急水源工程，今年争取向昆都仑水库蓄水，开工建设花圪台储水工程。加强国土资源保护，加快土地利用总体规划和矿产资源总体规划修编，集中整治各类违法违规用地采矿行为，促进土地节约集约利用和矿产资源科学利用。

　　（七）做好财税金融工作，更好地服务经济社会发展。抓住国家继续实施积极财政政策和适度宽松货币政策的机遇，进一步加大财税金融工作力度，充分发挥财税金融调节功能，为经济社会发展提供有力支撑。

　　全面增强财税保障能力。落实好扶持企业发展的政策措施，加大财源培植力度，壮大现有财源，培育后续财源，千方百计增加财政收入，确保实现全年财政收入目标。坚持依法治税，健全重点税源监控体系，突出抓好重点产业、重点企业的税收征管，确保应收尽收。强化公共财政职能，加强财政预算管理。优化财政支出结构，集中财力求发展、促和谐、办大事，努力提高财政资金使用效益。坚持勤俭办一切事业，继续大力压缩一般性开支，严格控制会议、差旅、出国、接待等公务费用。严肃财经纪律，加强对重点项目、社会保障等重点支出的审计监督和财政监督，严肃查处违法违纪行为。

　　提高金融服务水平。围绕建设金融大市、金融强市的目标，改善金融生态环境，扩大金融开放，增强金融业发展活力。通过强化银企对接、构建融资平台等措施，推动金融机构更多地支持我市产业发展和项目建设。改善中小企业融资服务，解决中小企业融资难问题。完善农村牧区金融体系，发展村镇银行，加大金融对“三农三牧”工作的支持力度。大力发展资本市场，推动企业上市融资，提高上市公司质量。推动包商银行加快发展。完善金融招商政策，加快金融商务区建设，力争华夏银行、内蒙古银行和光大银行年内入驻，争取更多金融机构落户包头。加快社会信用体系建设，形成有效的金融监管、风险控制及信用评价体系，增强金融服务地方经济发展能力。

　　（八）坚持统筹发展，促进社会事业全面进步。在推动经济平稳较快增长的同时，更加注重发展各项社会事业，不断满足人民群众对社会公共服务的需求。

　　优先发展教育事业。加快教育强市建设进程，加大教育投入，实施城乡一体的学校布局调整，推进义务教育学校办学条件标准化建设，促进基础教育均衡发展。优化课程体系和教学内容，切实减轻中小学生课业负担，努力推进素质教育。加快实施中小学校舍安全工程，全年完成180万平方米建设任务，占全部应改造面积的60%。大力推进职业教育，职业教育基地今年完成80万平方米建设任务。加强教师队伍建设，切实提高教师特别是农村牧区中小学教师素质和待遇。优先重点发展民族教育。扶持和规范民办教育，加强特殊教育、社区教育和继续教育。积极支持高等教育，促进高等院校更好地服务地方经济社会发展。加强组织领导，协同有关机构高水平举办2010年世界中学生排球锦标赛，积极筹备2011年全国中学生运动会。

　　增强医疗卫生服务能力。继续加强城市社区卫生工作和县乡村三级医疗卫生服务网络建设，加快实施土右旗医院、达茂旗医院病房楼和4个乡镇中心卫生院业务楼项目。完善疾病预防控制体系，做好重大传染病防控工作。深入开展国家卫生城市创建活动，全面提升城乡医疗卫生工作质量和管理水平。巩固和发展新型农村牧区合作医疗成果，实施新农合市级统筹，将新农合筹资标准提高到每人每年160元，高于全国和自治区平均水平，保障参合农牧民平等受益。深入推进“健康包头行动”，积极开展“一杯奶”生育关怀行动。培养农村牧区和社区卫生人才，建设市级临床医学领先学科及重点专科20个。积极筹建大型综合性医院，提高我市医疗服务水平。

　　推进文体广电事业繁荣发展。不断完善基础文化设施，推进基层文化室和综合文化站建设，完成文化信息资源共享工程基层网络建设。开工建设燕家梁遗址博物馆，建设南郊新型生态文化旅游区。高度重视培育文化创意产业，加快实施传媒大厦工程。办好鹿城文化艺术节等节庆活动，打造一批各具特色的文化品牌。加大文化遗产保护力度，推进非物质文化遗产保护传承工作。加强文化市场和新闻出版管理，营造健康繁荣的文化环境。大力发展文化产业，规划建设文化产业园区。逐步构建以文化旅游、影视动漫、文博会展等为重点的文化产业体系，提高文化产业占地区生产总值的比重。推进农村牧区广播电视微波无线覆盖工程，新建3座无线数字传递中继站，新增100个数字电视村。不断完善体育设施，规范体育市场管理，引进高水平赛事，全面推进体育产业化进程。新体育场、新体育馆今年要投入使用，开工建设市乒羽馆、游泳馆。组团参加自治区第十二届运动会和第七届少数民族传统体育运动会。深入贯彻《全民健身条例》，积极开展丰富多彩的全民健身活动，加快建设体育大市。

　　进一步提高各项社会事业发展水平。编制完成“十二五”规划。做好第六次全国人口普查工作。加强双拥工作，争创全国双拥模范城“七连冠”。认真实施国家和自治区妇女儿童发展纲要。重视残疾人事业发展，进一步完善残疾人服务设施。认真执行人口和计划生育政策，提高人口发展质量。加强老龄工作，推动社会化养老服务体系建设。全面发展审计、外事、侨务、地震、气象、档案、史志等各项事业。发挥工会、共青团、妇联等群团组织以及行业协会作用。

　　（九）努力保障和改善民生，维护好人民群众的根本利益。大力实施民生工程，切实解决人民群众关注的切身利益问题，努力让人民群众更多地享受改革发展成果。

　　突出抓好就业工作。加快构建覆盖城乡的就业服务网络，进一步落实和完善财政贴息、税费减免等支持创业、扶持就业政策，小额担保贷款发放力争达到2.4亿元。积极创建国家首批创业型城市，推进创业孵化园区建设试点工作，发挥好示范作用。有计划地培育和发展劳动密集型中小企业，吸纳更多人员就业。高度重视大中专毕业生和新增劳动力就业，鼓励支持高校毕业生在非公企业就业、自主创业和到城乡基层工作。实施积极就业政策，大力发展家政服务业，拓宽就业渠道。全年实现城镇新增就业5万人，下岗失业人员再就业2.1万人，其中，就业困难人员实现就业0.54万人。

　　不断完善社会保障体系。进一步扩大社会保险覆盖范围，积极推进中小企业、民营企业和个体工商户参保，做好城乡居民养老保险和城镇居民医疗保险工作，力争参保率分别达到50%和90%左右。进一步提高企业退休人员养老金待遇，月人均增加养老金要高于全区标准。逐步提高医疗保险保障水平。提高城乡居民最低生活保障标准。城镇居民低保标准人均每月提高30元。农牧民低保标准提高300元，达到每人每年1800元；低保补助水平提高120元，达到每人每年1300元。环卫一线清洁工人月工资再提高200元，达到1000元。提高社区专职干部待遇。高度关注特殊群体、特困群体、优抚群体的生活保障，做好大病医疗和教育、司法等各项社会救助工作。加强社会保险基金监管，确保基金安全、规范、高效运行。

　　不折不扣办好惠民实事。2010年，市委、市政府将围绕社会保障、公共服务、改善环境等内容，继续为人民群众兴办20件38项好事实事。在社会保障方面，要进一步加大财政投入力度，提高城镇职工、居民医疗保险最高支付限额和报销比例，提高“五保户”供养标准，提高城镇低保家庭取暖补贴标准，对困难家庭大中专学生进行资助等；在公共服务方面，要扩建包头市扶贫医院病房楼，通过推广使用IC卡优惠和方便居民乘坐公交车，完善天然气基础设施，建设100公里通村公路，等等。我们将以这些好事实事为重要抓手，把更多的人力、物力、财力投入到保障和改善民生上来，更多地为人民群众谋福祉，更多地让人民群众得实惠。

　　（十）加强精神文明和民主法制建设，全力维护社会和谐稳定。着力提高城乡居民文明素质和社会文明程度，强化社会管理工作，激发全民凝聚力和创造力，为全市改革发展营造良好的社会环境。

　　巩固和发展文明城市创建成果。以建设社会主义核心价值体系为根本，坚持贴近实际、贴近生活、贴近群众，继续深入开展精神文明创建活动。坚持以城带乡，构建城乡一对一结对共建网络，提高农村牧区精神文明建设水平。培养社会工作人才，扎实推进和谐社区建设，建成70个社区服务中心。强力净化社会文化环境，促进和保护未成年人健康成长。健全完善精神文明建设长效机制，争创“全国文明城市标兵”。

　　切实加强民主法制建设。自觉接受市人大及其常委会的法律监督和工作监督，自觉接受市政协的民主监督，认真听取民主党派、工商联、无党派人士和各人民团体的意见，办好人大代表建议和政协提案。推进政务、厂务、村务公开和企事业单位办事公开，畅通民主渠道，保障人民群众的知情权、参与权、表达权、监督权。牢牢把握各民族共同团结奋斗、共同繁荣发展主题，巩固和发展平等团结互助和谐的社会主义民族关系。继续抓好法律宣传和普法教育工作，进一步提高全民法治观念和法律素质，加快依法治市进程。规范司法行为，完善法律援助，加强仲裁工作，促进社会公平正义。

　　全面推进平安包头建设。完善应急管理体制机制和预案体系，加强气象防灾减灾和应对气候变化能力建设，提高保障公共安全和处置突发事件能力。坚持以治大隐患、防大事故为重点，着力加强对重大危险源的监控和重大安全隐患的整改，深入推进安全生产专项整治和质量标准化建设，坚决防止重特大事故发生。建立健全保障食品药品安全的责任体系，加强日常监管和专项整治，让群众购买到安全、放心的食品药品。做好群众来信来访、法律服务、法律援助和人民调解工作，完善多元化纠纷解决平台建设和工作机制，健全和完善领导定期接访、干部下访长效机制，及时化解各类社会矛盾。大力支持边防建设。加强社会治安综合治理，健全完善治安防控机制，依法严厉打击各类违法犯罪活动，保障人民生命财产安全。

　　各位代表，我们承担的工作艰巨而繁重，各级政府机构和工作人员必须切实加强自身建设，推进管理创新、服务创新、工作创新，不断适应新形势新任务的要求，为经济社会发展提供坚强保障。我们将进一步完善机制，确保中央、自治区重大决策部署贯彻落实，确保各项工作任务全面完成。建设学习型政府，大力弘扬求真务实、开拓创新之风，密切联系群众，加大督查力度，提高行政效能。认真落实党风廉政建设责任制，严格执行领导干部廉洁自律的各项规定。健全完善惩治和预防腐败体系，加强执法监察、廉政监察和效能监察，开展专项治理，坚决纠正损害群众利益的不正之风。加强公务员队伍建设，努力树立廉洁、务实、高效的政府形象。

　　各位代表，站在新的起点上，抢抓新机遇，实现新跨越，这是时代的呼唤，人民的重托。让我们更加紧密地团结在以胡锦涛同志为总书记的党中央周围，高举中国特色社会主义伟大旗帜，坚持以科学发展观为统领，继续团结拼搏，开拓创新，为建设经济繁荣、政治稳定、社会和谐、生态优美、人民幸福的科学发展示范地区而努力奋斗！
